# The backover threat: adopting thermal imaging infrared sensor for agricultural safety

**DOI:** 10.5249/jivr.v15i2.1847

**Published:** 2023-07

**Authors:** Bryan P Weichelt, David C. Schwebel, Serap Gorucu

**Affiliations:** ^*a*^ National Farm Medicine Center, Marshfield Clinic Research Institute, Marshfield, WI; National Children’s Center for Rural and Agricultural Safety and Health, Marshfield, Wisconsin, USA.; ^*b*^ Department of Psychology, University of Alabama at Birmingham, Birmingham, AL, USA.; ^*c*^ Department of Agricultural and Biological Engineering, University of Florida, Gainesville, Florida, USA.

We never want to think about losing the love of our life, someone we live and work alongside for decades, someone with whom we share hopes and dreams. Yet just such an event occurred February 23, 2015.^1^ A Wisconsin farm couple was doing chores like any other day, just as they had done hundreds of times before. The husband was operating a skid loader, a machine widely known to limit the operator’s field of vision. Just behind the skid loader, and as the machine was backing up, his wife slipped on ice. The husband drove over his wife. She was killed. 

Run over incidents are a leading event causing worker and bystander fatalities on farms, construction sites, and military training environments, occurring far too often in both occupational and non-occupational settings. In 2007 alone, 221 people were killed in the United States (US) when vehicles backed over them, and 99 of those (45%) were children under the age of 14. A total of 14,000 people, including 2,000 children, were seriously injured in the same manner.^2^ According to the US Bureau of Labor Statistics, 673 workers died from backover incidents from 2011 to 2020.^3^ It was reported that 137 bystander employees were injured (36 fatal, 101 nonfatal) in a skid steer related incident between 2015 and 2020.^4^ These numbers do not include non-working bystanders, such as young children or visitors in agricultural work environments.^5,6^ Heartbreakingly, each of these incidents was likely preventable. 

Old and new equipment alike can be equipped with technology to detect loved ones, workers, bystanders, visitors, children, pets, or livestock. Policy can be enacted to require safety technology in new equipment and machinery. While some progress around these kinds of tragic incidents has occurred, challenges remain. Advances in relevant technologies like built-in smart sensors and machine learning (also referred to as artificial intelligence) have increased passenger vehicle safety. Similarly, some construction equipment and vehicles are equipped with backup alert systems to warn bystanders, while proximity sensors can alert operators about close objects.^7,8^ These steps parallel what is now routine manufacturing practice in new motor vehicle production, with radar or ultrasonic sensors embedded into the rear or sides of the vehicle.^9^

However, skid steers present a unique challenge. On many farm and ranch operations, skid steers are used in very tight quarters, with walls, fencing, and other objects in close proximity to where the operator needs to maneuver the machine. Thus, standard back-up beeping alarms or sensors widely available on other types of vehicles have not been extensively adopted on skid steers, because they would frustrate users and fail to help prevent injuries and fatalities.

Infrared technology, which uses electromagnetic radiation to warn operators of live obstacles, provides a possible solution. Unlike alternatives that either allow operators to see physical obstacles or warn them of the presence of obstacles based on radar or ultrasonic technology, infrared technology uses electromagnetic radiation, undetectable to humans, to locate and respond to heat sources such as living bodies. This technology has existed for decades, with applications across military, police, emergency medical services (EMS), and construction. Technology transfer to agriculture has been slow, however, especially for health and safety applications that could prevent injury to workers and bystanders. If applied broadly, this technology could change skid steer safety, because it responds only when heat sources are present rather than when any obstacle is present. Thus, infrared offers a major advantage over alternatives: it alerts drivers only of the presence of living beings (e.g., children, pets, or livestock) who produce heat in the driver’s blind spot. Infrared is not limited by tight quarters and close proximity to nonliving barriers like walls and fencing. 

From a technical perspective, infrared technology relies on thermal remote sensing, or thermal imaging. Described as “the branch of remote sensing that deals with the acquisition, processing and interpretation of data acquired primarily in the thermal infrared region of the electromagnetic spectrum,” other agricultural production practices have utilized this imaging, including nursery management, soil health monitoring, disease and pathogen detection, and livestock management.^10-12^ Thermal imaging is also broadly employed to locate humans in military and search and rescue applications.^13^ Most recently, driven by the COVID-19 pandemic, the technology advanced rapidly to monitor body temperature for healthcare and public health objectives.^14,15^ Application of infrared technology to detect humans and animals in agricultural settings through machinery- or vehicle-mounted applications is a logical next step. 

Alternatives to infrared technology do exist. Application of radar-based sensors offers the advantage of low cost. Relying on detection of movement rather than heat, radar overcomes the limitation of false alarms from non-dangerous heat sources such as machinery, but it fails in tight quarters.^16^ Radar has begun to make its way into agricultural and construction worksites; the Built Robotics’ line of semi-autonomous equipment retrofits, for example, is built with radar sensor technology from Inxpect.^17,18^

Timely implementation of infrared technology could save lives. We propose two pathways to broad implementation of infrared technology on skid steers, and they would likely be most effective when jointly and swiftly initiated. First, policymakers could mandate that detection technology become standard issue among skid steers, tractors, and other equipment sold in the US (or other country’s) market, similar to US requirements for standard backup sensor/camera systems on automobiles. Second, owners of existing systems might retrofit their machines with sensor systems to protect safety, especially if the costs were rebated in a system comparable to ROPS installation on tractors. Even with joint and swift institution of those two pathways, it may take decades to have widely-distributed and implemented new obstacle-detecting sensors on skid steers operating on farms. Nevertheless, we need to start somewhere, and each installation reduces risk of tragic injuries. There were approximately 2.1 million farms in the US in 2021.^19^ More than 90% of those farms were small businesses or hobby farms, with the number of “lifestyle” farms increasing dramatically in recent years. In fact, experts anticipate increased numbers of people moving to country lifestyles, and, therefore, increased demand for smaller farm equipment.^20^

These small farms, and most US farms, have little regulatory oversight at the state or federal levels in terms of worker or child safety. Childcare options are slim and costly, and farm worksites are fraught with hazards.^21,22^ As the grieving husband on a Wisconsin farm sadly learned, skid steers’ features and structural components often lead to poor operator visibility. Today’s technology can address this hazard, and we urge stakeholders to study and apply infrared or other effective obstacle-detecting sensors on self-propelled machinery like skid steers. Lives can be saved. The cost to develop, implement and install outweighs the cost of ignoring the problem – the cost of a human life.
